# Tongue diagnosis indices for upper gastrointestinal disorders: Protocol for a cross-sectional, case-controlled observational study

**DOI:** 10.1097/MD.0000000000009607

**Published:** 2018-01-12

**Authors:** Tzu-Chan Wu, Keng-Liang Wu, Wen-Long Hu, Jer-Ming Sheen, Cheng-Nan Lu, John Y. Chiang, Yu-Chiang Hung

**Affiliations:** a Department of Chinese Medicine; b Division of Hepatogastroenterology, Kaohsiung Chang Gung Memorial Hospital and Chang Gung University College of Medicine; c Fooyin University College of Nursing; d Kaohsiung Medical University College of Medicine; e Department of Computer Science & Engineering, National Sun Yat-sen University; f School of Chinese Medicine for Post Baccalaureate I-Shou University, Kaohsiung, Taiwan.

**Keywords:** automatic tongue diagnosis system, panendoscopy, traditional Chinese medicine, upper gastrointestinal disorders

## Abstract

**Background:**

Upper gastrointestinal disorders are common in clinical practice, for example, gastritis, peptic ulcer disease, and gastroesophageal reflux disease. Panendoscopy or upper gastrointestinal endoscopy is viewed as the primary tool for examining the upper gastrointestinal mucosa, and permitting biopsy and endoscopic therapy. Although panendoscopy is considered to be a safe procedure with minimal complications, there are still some adverse effects, and patients are often anxious about undergoing invasive procedures. Traditional Chinese medicine tongue diagnosis plays an important role in differentiation of symptoms because the tongue reflects the physiological and pathological condition of the body. The automatic tongue diagnosis system (ATDS), which noninvasively captures tongue images, can provide objective and reliable diagnostic information.

**Methods:**

This protocol is a cross-sectional, case-controlled observational study investigating the usefulness of the ATDS in clinical practice by examining its efficacy as a diagnostic tool for upper gastrointestinal disorders. Volunteers over 20 years old with and without upper gastrointestinal symptoms will be enrolled. Tongue images will be captured and the patients divided into 4 groups according to their panendoscopy reports, including a gastritis group, peptic ulcer disease group, gastroesophageal reflux disease group, and healthy group. Nine primary tongue features will be extracted and analyzed, including tongue shape, tongue color, tooth mark, tongue fissure, fur color, fur thickness, saliva, ecchymosis, and red dots.

**Objectives:**

The aim of this protocol is to apply a noninvasive ATDS to evaluate tongue manifestations of patients with upper gastrointestinal disorders and examine its efficacy as a diagnostic tool.

## 1 Introduction

Upper gastrointestinal disorders associated with a range of troublesome symptoms are common in clinical practice, such as gastritis, peptic ulcer disease (PUD), and gastroesophageal reflux disease (GERD), among others. The most common symptoms of upper gastrointestinal disorders are abdominal pain or epigastric pain, and heartburn.^[1]^ It can be estimated that more than half of the world's population suffers from gastritis to some degree.^[2]^ It has also been reported that the annual incidence of PUD ranges from 0.10% to 0.19% for physician-diagnosed PUD,^[3]^ and that the prevalence of GERD is estimated to be 18.1% to 27.8% in North America, 8.8% to 25.9% in Europe, and 2.5% to 7.8% in East Asia.^[4]^ However, these may be underestimates due to the absence of symptoms in some cases and undiagnosed occurrences. If individuals ignore the warning signs or their symptoms are not properly diagnosed and managed, there can be severe complications. For example, patients with PUD are at risk for gastroduodenal hemorrhage or perforation, GERD is associated with Barrett esophagus, and chronic gastritis may be related to gastric cancer.

Panendoscopy, also referred to as upper gastrointestinal endoscopy and esophagogastroduodenoscopy (EGD), is the primary tool used for examining the upper gastrointestinal mucosa, and permits biopsy and endoscopic therapy.^[1]^ Panendoscopy is considered to be a safe procedure with minimal complications. However, there are still several risks associated with this procedure. The most frequent complications are associated with therapeutic interventions and include perforation, cardiac, or respiratory complications related to underlying comorbidities, and adverse effects from anesthesia.^[5]^ In addition, anxiety about undergoing invasive procedures is a serious problem in some patients. High levels of anxiety may lead to incomplete procedures, a higher chance of complications, or patients refusing endoscopy.^[6]^

Diagnosis in traditional Chinese medicine (TCM) is based on 4 procedures, observation, smelling or listening, inquiry, and palpation. Tongue diagnosis plays an important role in inspection and helps to differentiate between symptoms. The tongue is considered to reflect the physiological and pathological condition of the body, as well as the degree and progression of disease, through the meridians that connect the tongue to the internal organs.^[7,8]^ However, the result of tongue diagnosis often depends on subjective judgments and environmental factors.^[9]^ To obtain objective and quantitative diagnosis, many computerized tongue diagnosis systems have recently been developed.^[10]^ Several studies have used computerized tongue analysis to evaluate the relationship between tongue manifestations and various diseases, including rheumatoid arthritis,^[11]^ breast cancer,^[12,13]^ type 2 diabetes,^[14]^ metabolic syndrome,^[15]^ eczema,^[16]^ and dysmenorrhea,^[17]^ but seldom has the research focused on upper gastrointestinal disorders. The automatic tongue diagnosis system (ATDS) has shown high consistency and can provide objective and reliable information and analysis of tongue features, facilitating doctors in making effective observations and diagnoses of specific diseases.^[18]^

The objectives of this protocol are to apply the noninvasive ATDS to evaluate tongue manifestations in patients with upper gastrointestinal disorders, and to provide valuable information for clinical doctors, which can be used to facilitate the early detection and diagnosis of upper gastrointestinal disorders, to analyze the current status of patients, and to dynamically schedule treatment plans.

## 2 Methods

### 2.1 Ethics approval

This protocol has been reviewed and approved by the Institutional Review Board of the Chang Gung Medical Foundation (IRB no. 104-4725B). The protocol identification number at https://clinicaltrials.gov is NCT03258216. This study is conducted in accordance with the principles of the Declaration of Helsinki. Written informed consent will be obtained from all patients before enrollment. Personal information about potential and enrolled participants will be collected, shared, and maintained in an independent closet in order to protect confidentiality before, during, and after the trial.

### 2.2 Participants

Patients will be recruited from the outpatients of the Department of Hepatogastroenterology of Kaohsiung Chang Gung Memorial Hospital (KCGMH) in Kaohsiung, Taiwan.

Patients will be eligible if they satisfy the following criteria: age over 20 years old; with upper gastrointestinal symptoms, or who without symptoms and visit clinic for health examination; meet the criteria for examination via endoscopy; volunteered to join this research and signed the institutional review board agreement. Both men and women will be enrolled.

Patients with any of the following conditions will be excluded: hypertension, diabetes, hepatitis, or other systemic diseases; pregnancy; acute infection; cognitive impairment; unable to protrude the tongue stably; risk of temporomandibular joint dislocation.

### 2.3 Study design

This protocol is a cross-sectional, case-controlled observational study investigating the usefulness of the ATDS in clinical practice by examining its efficacy as a diagnostic tool for upper gastrointestinal disorders.

**Figure 1:**
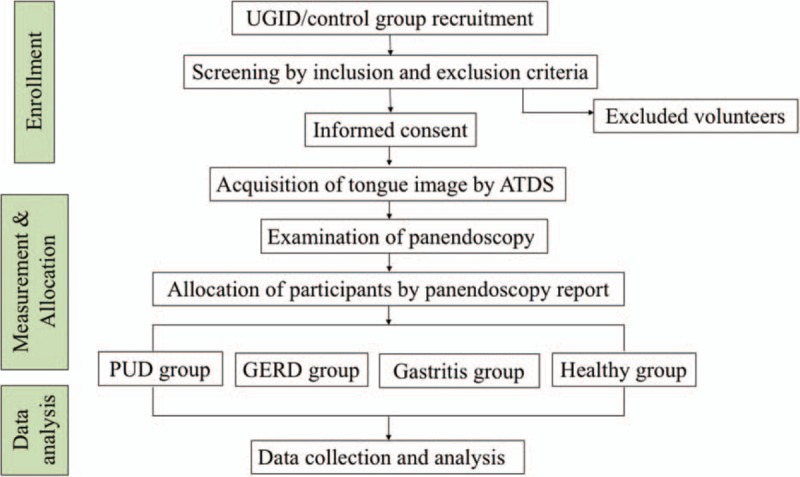
Flow chart of the study design. ATDS = automatic tongue diagnosis system; GERD = gastroesophageal reflux disease; PUD = peptic ulcer disease; UGID = upper gastrointestinal disease.

After giving their consent, participants will undergo tongue image capturing using the ATDS. The ATDS examination will be performed under constant environmental conditions and by the same educated operator. After capturing tongue image, participants accept panendoscopy which will be performed by a professional gastroenterology doctor. According to findings of panendoscopy, the participants will be allocated to the following 4 groups: a peptic ulcer disease group (PUD, including patients diagnosed with peptic ulcers), a gastroesophageal reflux group (GERD, including patients diagnosed with reflux esophagitis or gastroesophageal reflux), a gastritis group (GA, including patients diagnosed with gastritis), and a healthy group (H, including patients with negative results from the gastric endoscopy procedure). A flow chart of the trial design is presented in Fig. 1.

### 2.4 Intervention: automatic tongue diagnosis system

**Figure 2:**
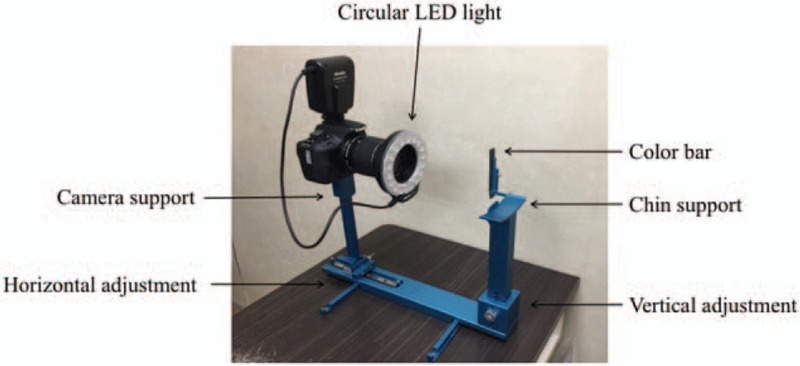
Components of the automatic tongue diagnosis system.

As shown in Fig. 2, the ATDS was developed to capture tongue images and reliably analyze tongue features. The system has 3 major functions: image capturing and color calibration, tongue area segmentation, and tongue feature extraction.

To reduce the influence of background surroundings, the ATDS is placed in a set location and the operator and the patient sit on fixed seats. To start, the well-trained operator adjusts the chin support horizontally and vertically in order to capture the whole tongue. Then, the patient protrudes their tongue and holds it relaxed and stable for about 5 seconds, allowing the operator to capture a tongue image.

The ATDS can automatically correct any lighting and color deviation caused by changes in background lighting using a color bar attached beside the chin support. After capturing, the tongue images are prepared by isolating the tongue region to eliminate any irrelevant sections of the image including the teeth, lower facial portions, or background surrounding the tongue. After this, feature identification and extraction can be carried out.

### 2.5 Outcome measures

#### 2.5.1 Primary outcome measures

Nine primary tongue features will be extracted from the ATDS as follows: tongue shape: small and thin, moderate, large, and fat; tongue color: slightly white, slightly red, red, dark red, and dark purple; tooth mark: includes number, average covering area, maximum covering area, minimum covering area, and organs corresponding to the covering area; tongue fissure: amount, average covering area, shortest length, and longest length; fur color: white, yellow, and dye; fur thickness: none, thin, thick; fur amount, average covering area, maximum covering area, minimum covering area, and organs corresponding to the covering area; saliva: includes total area and the amount of saliva (none, little, normal, excessive); ecchymosis: amount, average covering area, maximum covering area, minimum covering area, and organs corresponding to the covering area; red dots: number, average covering area, maximum covering area, minimum covering area, and organs corresponding to the covering area.

Feature identification will be further subdivided into 5 segments (spleen–stomach, liver-gall-left, liver-gall-right, kidney, and heart–lung area) according to the theory of traditional Chinese medicine.

#### 2.5.2 Secondary outcome measures

Panendoscopy reports written by professional gastroenterology doctors will be recorded.

### 2.6 Sample size

We calculated the sample size will be 517 with power = 0.9, alpha = 0.05, effect size convention *r* = 0.3, and an anticipated drop-out rate of 10%, using G∗Power 3.0.1.0 software which is download from http://www.gpower.hhu.de.

### 2.7 Data analysis

All statistical analyses will be performed using the SPSS statistical package program, version 17.0 (SPSS Inc., Chicago, IL). Chi-square tests will be applied for categorical data and Analysis of variance tests will be applied for continuous data. Logistic regression will be used to estimate the odds ratio and the probability of a binary response, based on one or more independent variables. *P*-values <.05 will be considered statistically significance.

### 2.8 Data monitoring

Data monitoring committee (DMC) is not needed because of this observational study.

## 3 Discussion

According to the theory of traditional Chinese medicine, the tongue is thought to be an outer manifestation of the status of the viscera, and can be divided to spleen–stomach area, liver–gall area, kidney area, and heart–lung area. The tongue coating is formed by “stomach-Qi” and the 5 organs (“Wu-Zang” in Chinese) are also supplied by the stomach.^[19]^ Therefore, the tongue, especially spleen–stomach area, and the tongue coating may reflect the status of the spleen and stomach. The results of this trial are expected to provide valuable evidence supporting the use of tongue diagnosis to evaluate the status of patients with upper gastrointestinal disorders, helping clinical doctors to identify potential problems, and implement proper management of these conditions.

There are several studies that discuss the relationship between gastrointestinal disorders and tongue characteristics such as tongue coating thickness, tongue coating microbiota, and metabolic markers, and tongue temperature. Kim et al^[20]^ utilized the tongue coating thickness on patients with fuctional dyspepsia to assess the availability of tongue diagnosis system. Sun et al^[19]^ found that changes in metabolic pattern and miroecological index of tongue coating were associated with chronic gastritis. Cheng et al^[21]^ reported that patients suffering from gastrointestinal disease with *Helicobacter pylori* have a higher tongue temperature but have no statistically significance between the control group. However, the proposed study will provide more details and evidence of the usefulness of tongue image analysis for the identification of upper gastrointestinal disorders. In addition, this method of tongue diagnosis could be utilized in clinical practice and education.

There are 4 groups in this trial, namely, the peptic ulcer disease group, the gastroesophageal reflux group, the gastritis group, and the healthy group. Gastritis, PUD, and GERD are all associated with mucosa injury, but with different clinical manifestations and pathologies. We are interested in studying the differences between them, which might be visible on tongue images, and in assessing the relationship between panedoscopic images and tongue diagnosis.

In conclusion, TCM tongue diagnoses are expected to serve as preliminary screening indices for upper gastrointestinal disorders.

## 4 Authors’ contributions

Y.C. Hung and J.Y. Chiang were responsible for the design and supervision of the study, and revision of the manuscript. T.C. Wu and K.L. Wu drafted the manuscript and undertook the trial registration. W.L. Hu, J.M. Sheen, and C.N. Lu participated in the revision of the manuscript and coordination of the study. Y.C. Hung and J.Y. Chiang designed the statistical plan. T.C. Wu and K.L. Wu participated in data acquisition. All authors read and approved the final manuscript.

## Acknowledgments

The authors would like to express our thanks to the other members of the research team who will participate in the application of this research protocol: Jun-Cheng Su and Ming-Zhi Lin.
